# Interclavicularis anticus digastricus muscle in a female body donor: a case report

**DOI:** 10.1007/s00276-021-02848-w

**Published:** 2021-10-09

**Authors:** M. K. Roesler, M. J. Schmeisser, S. Schumann

**Affiliations:** 1 Institute for Microscopic Anatomy and Neurobiology, University Medical Center of the Johannes Gutenberg-University, Mainz, Germany; 2 Focus Program Translational Neurosciences (FTN), University Medical Center of the Johannes Gutenberg-University, Mainz, Germany

**Keywords:** Sternum, Clavicle, Muscular variations, Pectoralis major muscle, Lateral pectoral nerve

## Abstract

**Background and objectives:**

Muscular variations of the ventral thoracic wall are generally common and of great clinical interest.

**Materials and methods:**

An unusual muscular variation of the ventral thoracic wall was observed and dissected in a West-European female body donor.

**Results:**

An interclavicularis anticus digastricus muscle was observed and studied. It originated from the manubrium sterni and inserted bilaterally to the clavicles. Both muscle bellies were interconnected by a tendon on the ventral surface of the manubrium sterni. The muscle was innervated by branches of the lateral pectoral nerve.

**Conclusions:**

The interclavicularis anticus digastricus muscle is a muscular variation of the ventral thoracic wall of unknown prevalence. This variation might be of clinical interest in orthopaedics and thoracic surgery. It is also a vulnerable structure during infraclavicular insertion of a subclavian vein catheter or fractures of the clavicle.

## Introduction

The ventral thoracic wall is built by various bones, muscles, and connective tissue. Multiple muscular variations of the ventral thoracic wall are known, and variations of pectoral muscles are said to be more frequent than variations of any other muscle group [4, 7] Variations include the partial or complete absence of muscles, additional muscle heads or bodies, unusual origins or insertions, fusion or fission of muscles or the existence of supernumerary muscles [11]. Knowledge of muscular variations is of clinical interest due to diagnostical and therapeutical intervention in this region.

Supernumerary muscles of the ventral thoracic wall (e.g. pectoralis quartus muscle, Langer's axillary arch, chondroepitrochlearis muscle, or sternalis muscle) [28] were classified by Huntington according to their topographical position into superficial supernumerary muscles, placed superficially to the pectoralis major muscle, and deep supernumerary muscles, located in the space between the pectoralis major and pectoralis minor muscles [15]. The interclavicularis anticus digastricus muscle (IADM) is a deep supernumerary muscle of the ventral thoracic wall and considered to be a special bilateral condition of the variant sternoclavicularis anticus muscle (SAM) [28].

## Materials and methods

**Fig. 1:**
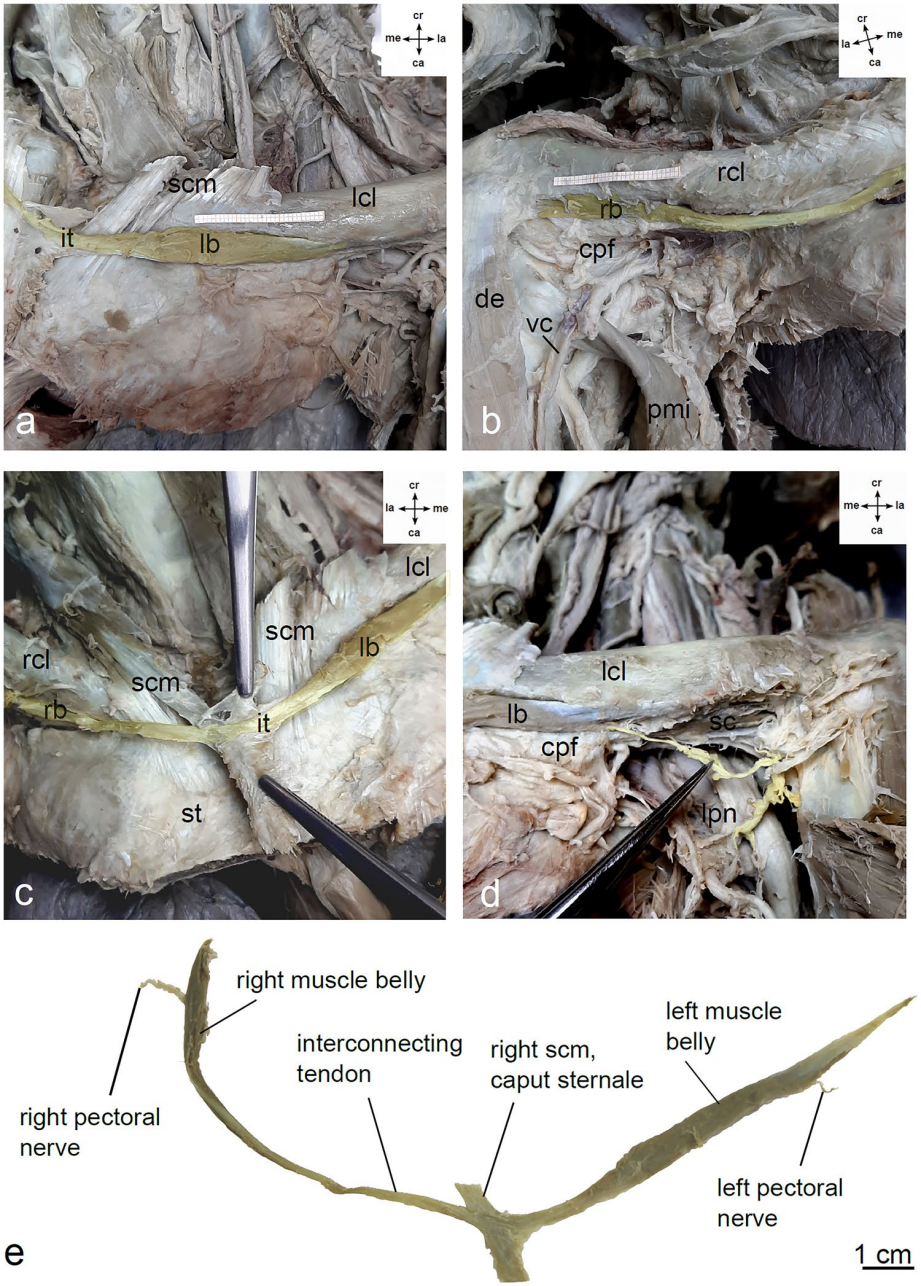
**a** Left muscle belly of the interclavicularis anticus digastricus muscle (IADM, highlighted in yellow). The left pectoralis major muscle was resected. Graph paper on the sternal end of the left clavicle indicates a length of 3 cm. **b** Right muscle belly of the IADM (highlighted in yellow). The right pectoralis major muscle was resected. Graph paper on the acromial end of the right clavicle indicates a length of 3 cm. **c** Interconnecting tendon (highlighted in yellow) on the ventral surface of the manubrium sterni. Connective tissue surrounding the tendon in forceps. **d** Branch from the left lateral pectoral nerve (in forceps, highlighted in yellow) to the left muscle belly of the IADM. **e** Resected IADM including the left and right muscle belly with the according nerves and interconnecting tendon. *cr* cranial, *ca* caudal, *la* lateral, *me* medial, *lcl* left clavicle, *rcl* right clavicle, *cpf* clavipectoral fascia, *de* deltoid muscle, *it* interconnecting tendon, *lb* left muscle belly, *lpn* left pectoral nerve, *rb* right muscle belly of the IADM, *mpi* pectoralis minor muscle, *sc* subclavius muscle, *scm* sternocleidomastoideus muscle, *st* sternum, *vc* cephalic vein

An unusual muscular variation was observed in a 90-year-old West-European female who died of gastric cancer. We lack any further clinical information. The woman was part of the body donation program of the Anatomical Institutes of the Johannes Gutenberg-University, Mainz, Germany. She donated her body voluntarily for medical education and research. The specimen had been fixated via arterial perfusion with formaldehyde and subsequent formaldehyde immersion within a humidity chamber. Dissection was performed during the routine dissection class for undergraduate medical students at the Institute for Microscopic Anatomy and Neurobiology in the winter term 2020/2021. For morphometrical analysis, the muscle was removed carefully. Measurement was performed using a calliper. Inkscape project (2020, Inkscape) was used to create the Fig. 1.

## Case report

During dissection of the ventral thoracic wall, we observed an IADM, a special bilateral condition of a SAM. This muscle originated from the ventral surface of the manubrium sterni. The muscle inserted bilaterally with one muscle belly each on the inferior surfaces of the acromial ends of the clavicles. The two muscle bellies showed an interesting asymmetry: on the left side, the muscle belly was spindle-shaped and lay beneath the medial third of the clavicle (Fig. 1a). It inserted with a thin tendon on the inferior surface of the acromial end of the clavicle. On the right side, the muscle belly was fan-shaped and attached directly (fleshy insertion) to the acromial end of the clavicle (Fig. 1b). On the ventral surface of the manubrium sterni, both muscular origins were interconnected by a tendon (Fig. 1c). This interconnecting tendon intercalated with the tendon of the sternal head of the right sternocleidomastoid muscle. Thin fibres of the lateral pectoral nerves reached the muscle bellies and entered at the inferior borders (Fig. 1d, e). The muscle bellies were separated from the subclavius muscle by a thick fascial layer of the clavipectoral fascia. Additionally, we reported an underdevelopment of the clavicular part of the pectoralis major muscle on both sides with an enlarged groove between the clavicular and sternocostal part on the left side and an enlarged deltopectoral groove on the right side. The pectoralis minor and subclavius muscle showed a normal morphology. No further variations of the ventral thoracic wall were observed. For morphometric data see Table 1.

**Table 1:** Morphometry of the interclavicularis anticus digastricus muscle

Interclavicularis anticus digastricus muscle	
Total length	19.5 cm
Left muscle belly	
Length	4.5 cm
Width	0.6 cm
Thickness	0.3 cm
Left insertion tendon	3.0 cm
Right muscle belly	
Length	5.5 cm
Width	0.4 cm
Thickness	0.2 cm
Right insertion tendon	0.0 cm (fleshy insertion)
Interconnecting tendon	
Length	6.5 cm
Width	0.2 cm
Thickness	0.05 cm
Neurovascular hilum (distance to the lateral end of the muscle belly)	
Left	2.6 cm
Right	1.1 cm
Clavicular part of pectoralis major muscle	
Left	2.9 cm
Right	4.0 cm
Interpectoral groove (maximum width)	
Left	0.9 cm
Right	0.0 cm (no groove)
Deltopectoral groove (maximum width)	
Left	0.0 cm (no groove)
Right	4.0 cm

## Discussion

Supernumerary clavicular muscles are numerous but uncommon. They were classified by Testut into four groups: 1. muscles sterno-chondro-scapulaires (sternochondroscapular muscles), muscles sterno-claviculaires (sternoclavicular muscles), muscles scapula-claviculaires (omoclavicular muscles), and muscles cleido-aponévrotiques (clavicle-fascial muscles) [27].

The sternoclavicularis anticus muscle (SAM, synonyms: sternoclavicularis muscle, sternoclavicularis anterior muscle, praeclavicularis medialis muscle) is a rare muscular variation of the ventral thoracic wall [6, 8, 9, 12–15, 20, 25, 26, 29, 32, 33]. This variation belongs to the sternoclavicular muscle group, classified by Testut [27]. It arises from the ventral surface of the manubrium sterni, the anterior sternoclavicular ligament and/or the cartilage of the first and second rib before it inserts to the inferior surface of the clavicle. The first description of a SAM was given by Wenzel Gruber in 1860. In this first study, this variation is described in two male adults and one male child [12]. According to Gruber, the prevalence of the SAM is between 2.44 and 3.33% [12, 13]. The length of this variation ranges between 0.47 and 13 cm [10].

In rare cases, a bilateral SAM was found [8, 9, 13–15, 25, 29]. The first description of a bilateral SAM was also given by Gruber [13]. In this study, he described two cases of bilateral SAM, one in a male adult, and one in a male child. Gruber distinguished between a bilateral SAM without an interconnecting tendon (two individual muscles) and an IADM with an interconnecting tendon, forming a digastric muscle [13, 15, 25]. According to this classification, our variation must be classified as an IADM. To our knowledge, data on the frequency of occurrence of this special condition are lacking. We were able to identify five cases of IADM in literature (three cases of Gruber [13, 14], one case of Dwight [9] and one case of Sakuma [25]). Unfortunately, the case descriptions of Clason [8], Huntington [15] and Umesue [29] do not allow a distinction between bilateral SAM and IDAM.

There is a great variety of other supernumerary clavicular muscles that must be distinguished from SAM and IDAM. Hubert Luschka first described a supraclavicular muscle (synonyms: sternoclavicularis superior muscle, supraclavicularis medialis muscle, supraclavicularis superior muscle) which connects the suprasternal ossicles and the interclavicular ligament with the superior surface of the clavicle. Bilateral forms of this muscle are known [16, 19]. In case of bilateral occurrence, the muscle bellies can be separated, fused, or connected by an interconnecting tendon [16, 19]. The supraclavicularis proprius muscle (synonym: tensor fasciae colli muscle) runs from the medial end of the clavicle to its lateral end. The muscle is connected to the deep lamina of the superficial cervical fascia [13, 23]. The sternoclavicularis posticus muscle (synonym: retroclavicularis muscle) connects the posterior surface of the manubrium sterni with the sternal ends of the clavicle [30]. The infraclavicularis muscle, first described by Testut, originates from the ventral surface clavicle and interests into the deltoid fascia [17, 27]. Bilateral forms of this variation have been described [31]. The praeclavicularis lateralis muscle (synonym: acromioclavicularis muscle) connects the lateral end of the clavicle with the acromion [13]. The subclavius posticus muscle (synonyms: sternoscapularis muscle, sternochondroscapularis muscle, scapulocostalis minor muscle) was discovered by Rosenmüller [18]. It originates from the sternum, the costal cartilage, or the first rib and inserts to the scapula [24]. This muscle can be fused with the subclavius muscle [21]. Its prevalence ranges between 1 and 7% [22]. The costoclavicularis muscle originates from the second rib and inserts into the inferior surface of the clavicle. Its prevalence is less than 1% [22].

In the present case, we could confirm that the IADM receives its nerve supply from small branches of the lateral pectoral nerves from the brachial plexus. The lateral pectoral nerves arise from the lateral cord of the brachial plexus and contain fibres from the cervical segments 5 to 7 [28].

The occurrence of a SAM or an IADM seems to coincidence with variations of the clavicular portion of the pectoralis major muscle. Variations of the pectoralis major muscle are common in general. Its most common variation is the absence of the sternocostal head [28]. Absence of the clavicular head is rare [7], although it occurs concomitantly with the occurrence of supernumerary muscles [6]. A SAM can be associated with other muscular anomalies like a sternalis muscle [6]. Interestingly, we did not observe morphological alterations of the pectoralis minor muscle and the subclavius muscle. This might be explained by the embryology of these muscles.

Since the IADM correlates with muscular variations of the pectoralis major muscle and is innervated by the same nerve, both muscles may share the same developmental origin. Embryologically, the pectoralis muscles are hypaxial muscles. The pectoralis blastema splits into a cranial part, which is the anlage of the clavicular head of the pectoralis major muscle, and a caudal part, which is the primordium of the pectoralis minor muscle and the sternocostal head of the pectoralis major muscle [3]. The subclavius muscle develops from a common anlage with the inferior belly of the omohyoid muscle [1, 2]. The SAM and IADM most likely develop together with the clavicular hear of the pectoralis major muscle from the cranial part of the pectoralis blastema.

There are only putative functions of the IADM. It may stabilize the sternoclavicular joint or lead to a weak protraction of the clavicle in the sternoclavicular joint. In case of common clavicular fracture (approximately 5.0% of all fractures), this muscle might influence the displacement of the bony fragments. The clavicular part of the pectoralis major muscle has an important role in arm flexion and medial rotation [5]. These functions might be affected in the present case due to the underdevelopment of the clavicular part of the pectoralis major.

In this case report, we used the terms sternoclavicularis anticus muscle and interclavicularis anticus digastricus muscle since these terms were introduced by Gruber in his original description of these variations [12, 13]. Although, since the term anticus is no longer used in anatomical terminology, we suggest establishing the terms sternoclavicularis anterior muscle, bilateral sternoclavicular anterior muscle (for the bilateral form without an interconnecting tendon) and interclavicularis anterior muscle (for the bilateral form with an interconnecting tendon).

## Conclusion

The interclavicularis anticus digastricus muscle is a rare muscular variation of the ventral thoracic wall of unknown prevalence. Physicians should be aware of this rare muscular variation during operative treatment of clavicle fractures or insertion of a subclavian vein catheter.

## Data Availability

Not applicable.
